# Interrupted time series analysis using the ARIMA model of the impact of COVID-19 on the incidence rate of notifiable communicable diseases in China

**DOI:** 10.1186/s12879-023-08229-5

**Published:** 2023-06-05

**Authors:** Qin Zhou, Junxian Hu, Wensui Hu, Hailin Li, Guo-zhen Lin

**Affiliations:** Department of disease control and prevention, Guangzhou Center for Disease Control and Prevention, No. 1 Qide Road, Baiyun district, 510440, 510440 Guangzhou, Guangzhou, Guangdong China

**Keywords:** Communicable diseases, COVID-19, Incidence rate, Interrupted time series analysis, Autoregressive integrated moving average models

## Abstract

**Background:**

The coronavirus disease 2019 (COVID-19) pandemic in China is ongoing. Some studies have shown that the incidence of respiratory and intestinal infectious diseases in 2020 decreased significantly compared with previous years. Interrupted time series (ITS) is a time series analysis method that evaluates the impact of intervention measures on outcomes and can control the original regression trend of outcomes before and after the intervention. This study aimed to analyse the impact of COVID-19 on the incidence rate of notifiable communicable diseases using ITS in China.

**Methods:**

National data on the incidence rate of communicable diseases in 2009–2021 were obtained from the National Health Commission website. Interrupted time series analysis using autoregressive integrated moving average (ARIMA) models was used to analyse the changes in the incidence rate of infectious diseases before and after the COVID-19 epidemic.

**Results:**

There was a significant short-term decline in the incidence rates of respiratory infectious diseases and enteric infectious diseases (step values of -29.828 and − 8.237, respectively), which remained at a low level for a long time after the decline. There was a short-term decline in the incidence rates of blood-borne and sexually transmitted infectious diseases (step = -3.638), which tended to recover to previous levels in the long term (ramp = 0.172). There was no significant change in the incidence rate of natural focus diseases or arboviral diseases before and after the epidemic.

**Conclusion:**

The COVID-19 epidemic had strong short-term and long-term effects on respiratory and intestinal infectious diseases and short-term control effects on blood-borne and sexually transmitted infectious diseases. Our methods for the prevention and control of COVID-19 can be used for the prevention and control of other notifiable communicable diseases, especially respiratory and intestinal infectious diseases.

In December 2019, an unknown pneumonia broke out in Wuhan China, which was proven to be caused by a novel coronavirus and named novel coronavirus pneumonia(coronavirus disease 2019, COVID-19) [1]. In January 2020, COVID-19 spread to the whole country. To prevent and control the epidemic, all provinces in China successively launched a public health emergency response, and many measures were implemented for the prevention and control of COVID-19[2]. As the epidemic was gradually controlled, all provinces gradually adjusted the public health emergency response level and established a normalized policy on the prevention and control of COVID-19.

The positive effects brought by the COVID-19 pandemic were improvements in residents’ awareness and practice measures of prevention and control, such as hand washing and mask wearing which had an influence on other infectious diseases [3]. Some studies have shown that the incidence of respiratory and intestinal infectious diseases in 2020 decreased significantly compared with previous years[3, 4]. However, previous studies directly compared the differences before and after the epidemic, and did not consider the seasonal or periodic features of infectious diseases. Moreover, previous studies did not consider long-term effects. Interrupted time series (ITS) is a time series analysis method proposed by Box and Tiao to evaluate the impact of intervention measures on outcomes and can control the original regression trend of outcomes before and after the intervention, compare the immediate level changes of outcomes, and evaluate the impact of intervention measures on outcomes in short-term and long-term dimensions [5, 6]. ITS is the strongest quasi-experimental design for analysing the impact of intervention measures on time series, and it is widely used in evaluating social policies, public health policies and environmental policies [7–9].

Most ITS analyses use piecewise linear regression, but require that the long-term trend of the outcome variable before and after the intervention be linear. The incidence rate of most infectious diseases has seasonal periodicity, which makes the time series of infectious disease incidence autocorrelated and unstable, and it is not suitable to use piecewise regression directly. Therefore, the seasonal periodicity of the incidence rates of infectious diseases needs to be controlled. The periodicity of the time series can be controlled by the autoregressive integrated moving average (ARIMA) model [10].

The ARIMA model is one of a series of time series analysis methods proposed by Box and Jenkins in the 1960s and is one of the common time series prediction models [5]. The ARIMA model is widely used in health and other fields. In the field of public health, the ARIMA model is often used to detect the outbreak of infectious diseases and predict the epidemic trend of the disease [11, 12]. In the ITS analysis, the ARIMA model controls nonstationarity or seasonality by capturing the time series trend in the sequence data, as well as controlling the autocorrelation of the sequence, which can well identify the periodicity and long-term trend of the data [13].

This study used ITS to analyse the impact of the COVID-19 epidemic on the incidence of infectious diseases in China, and the ARIMA model to control the periodicity of the incidence in the ITS analysis. This study provided a scientific assessment of the impact of COVID-19 prevention and control measures on other infectious diseases and a scientific basis for the prevention and control of infectious diseases in the postepidemic era in China.

## Materials and methods

### Data source

The incidence data on notifiable infectious diseases in China from 2009 to 2021 (excluding cases from Hong Kong, Macao, Taiwan and foreign countries) were from the Disease Control and Prevention Bureau of the National Health Commission website (http://www.nhc.gov.cn/jkj/)and the Journal of Disease Surveillance [14]. The population data were from the National Bureau of Statistics website http://www.stats.gov.cn/.

### Classification of nationally notifiable infectious diseases

According to China’s law on the prevention and control of infectious diseases (2020 revised version) [15], notifiable infectious diseases include 39 kinds that are divided into three categories: Class A,Class B and Class C (Table 1). As the number of Class A infectious diseases reported is very small every year, the analysis of this study combined Class A and Class B diseases.

According to the transmission route, 39 infectious diseases were divided into four categories (except for neonatal tetanus) (Table 2): intestinal infectious diseases, respiratory infectious diseases, natural focal and insect-borne diseases, and blood-borne and sexually transmitted diseases.

**Table 1:** Classification of nationally notifiable infectious diseases in China

Classification	Diseases
Class A	Pestis, cholera
Class B	Severe acute respiratory syndrome (SARS), human immunodeficiency virus/acquired immunodeficiency syndrome (HIV/AIDS), viral hepatitis, poliomyelitis, human infection with highly pathogenic avian influenza, measles, epidemic haemorrhagic fever, rabies, epidemic encephalitis B, dengue fever, anthrax, bacterial and amoebic dysentery, tuberculosis, typhoid and paratyphoid, epidemic cerebrospinal meningitis, pertussis, diphtheria, neonatal tetanus, scarlet fever, brucellosis, gonorrhoea, syphilis, leptospirosis Schistosomiasis, malaria, human infection with H7N9 avian influenza
Class C	Influenza, mumps, rubella, acute haemorrhagic conjunctivitis, leprosy, typhus, Kala azar, hydatidosis, filariasis, other infectious diarrhoea, hand, foot and mouth disease

**Table 2:** Classification of nationally notifiable infectious diseases by different transmission route in China

Transmission route	Diseases
Intestinal infectious diseases	Cholera, viral hepatitis other than hepatitis B and C, poliomyelitis, bacterial and amoebic dysentery, typhoid, paratyphoid, other infectious diarrhoea, hand, foot and mouth disease, acute haemorrhagic conjunctivitis
Respiratory infectious diseases	Severe acute respiratory syndrome (SARS), measles, tuberculosis, epidemic cerebrospinal meningitis, pertussis, diphtheria, scarlet fever, human infection with H7N9 avian influenza, influenza, mumps, rubella, leprosy
Natural focal and insect-borne diseases	Pestis, human infection with highly pathogenic avian influenza, epidemic haemorrhagic fever, rabies, epidemic encephalitis B, dengue fever, anthrax, brucellosis, leptospirosis, schistosomiasis, malaria, typhus, Kala azar, hydatidosis, filariasis
Blood-borne and sexually transmitted diseases	Human immunodeficiency virus/acquired immunodeficiency syndrome (HIV/AIDS), hepatitis B, hepatitis C, gonorrhoea, syphilis

### Statistical analysis

The incidence rate was used to describe the changes in infectious diseases before and after the COVID-19 epidemic. Incidence rate = (number of patients in the year/average population in the year) × 100,000.

The change in the incidence rate= [(the average incidence rate from 2020 to 2021 – the average incidence rate from 2009 to 2019)/the average incidence rate from 2009 to 2019] × 100%.

Since H7N9 avian influenza and hepatitis D were added to the nationally notifiable infectious disease list in November 2013 and January 2016, respectively, only the average incidence rates of H7N9 avian influenza in 2014–2019 and hepatitis D in 2016–2019 were calculated. Influenza A (H1N1) was moved from Class B to Class C in Novermber 2013 and included as influenza. Therefore, the incidence rate of influenza A (H1N1) before Novermber 2013 was included in the incidence rate of influenza.

### ITS-ARIMA modeling building

Taking incidence rate as the dependent variable and time as the independent variable, the step value (short-term-level variable) and ramp value (slope of the trend variable) were introduced as independent variables to construct the ITS. An ARIMA model based on the ITS was established for the time series of incidence rates from January 2009 to December 2021. The model was ARIMA (p, d, q) × (P, D, Q) s, where p, d and q represent the autoregressive order, difference order and moving average order of the time series, respectively. P, D and Q represent the seasonal autoregressive order, seasonal difference order and seasonal moving average order, respectively. s represents the seasonal cycle.

The model parameters included step term and ramp term: the step term represented the short-term change in the incidence rate after the intervention, which was the difference between the actual observed value and the predicted value after the epidemic. The ramp term indicated the slope change in the incidence rate, which was the difference in the slope after the intervention and before the epidemic. Moreover, the incidence rate without the intervention from February 2020 to December 2021 was predicted to show the change in the incidence rate after the intervention according to the model. The model was built by R 4.1.0 software.

### Interpretation

**Fig. 1:**
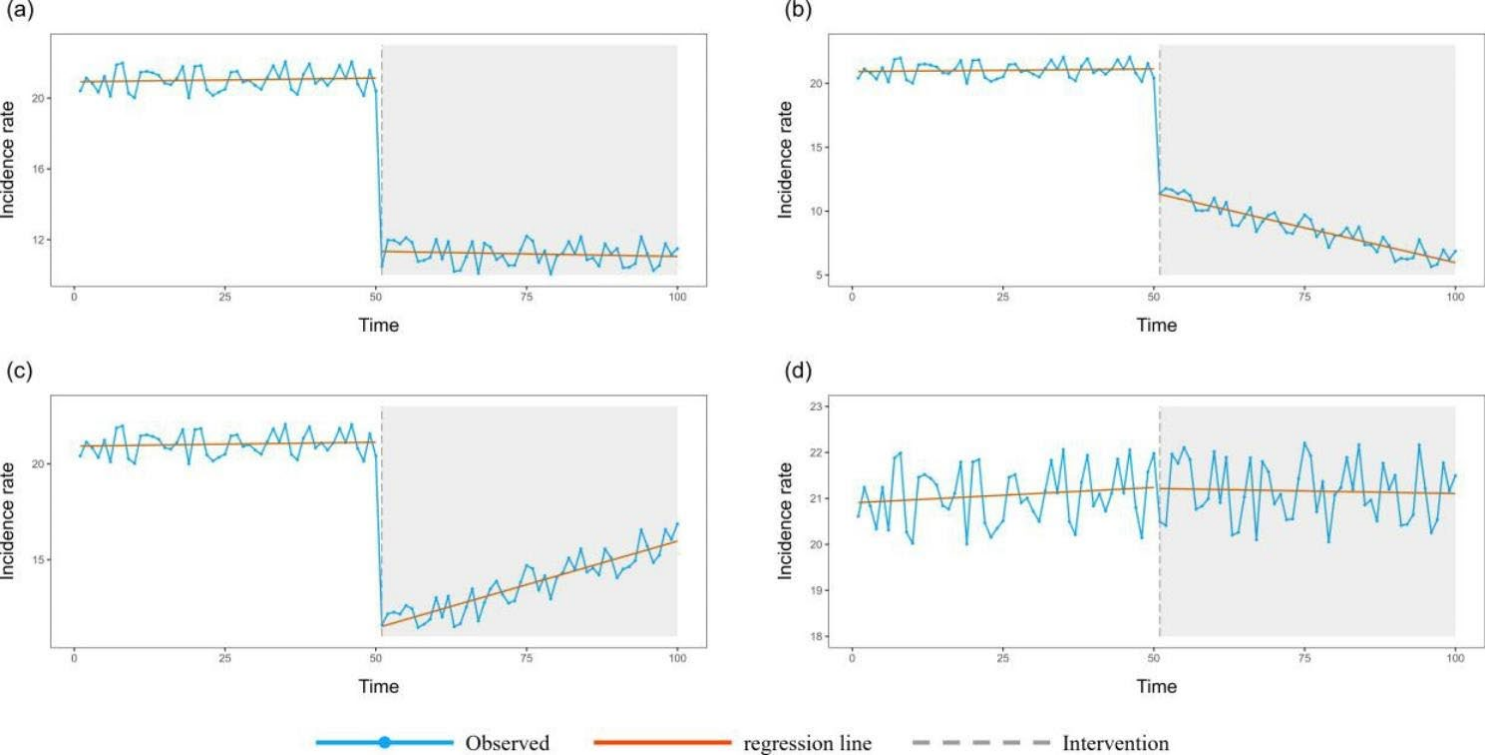
The four possible expected results from the ITS-ARIMA model

In this study, the step and ramp of the ITS-ARIMA model were used to represent the short-term and long-term changes in the incidence rates of infectious diseases after the COVID-19 epidemic. The expected results were as follows: 1. Step < 0, no significant change in the ramp value(Fig. 1A): The incidence of infectious diseases significantly decreased temporarily after the COVID-19 epidemic and remained at a low level in the long term; 2. step < 0 and ramp < 0 (Fig. 1B): The incidence of infectious diseases significantly decreased temporarily after the COVID-19 epidemic and continued to decrease in the long term; 3. step < 0 and ramp > 0 (Fig. 1C): The incidence of infectious diseases significantly decreased temporarily after the COVID-19 epidemic and showed a recovery trend to the previous level in the long term; and 4. there was no significant change in the step or ramp values (Fig. 1D), which meant that the incidence rate did not change significantly either in the short term or long term after the COVID-19 epidemic.

## Results

### Incidence rate of nationally notifiable infectious diseases in China from 2009 to 2021

From 2020 to 2021, a total of 13,161,948 cases of notifiable infectious diseases other than COVID-19 were reported nationwide. The average incidence rate in the two years was 466.2/100,000 people, a decrease of 16.70% compared with the average rate from 2009 to 2019. A total of 6,408,089 cases of classes A and B infectious diseases were reported, with an incidence rate of 227.0/100,000 people, a decrease of 16.04% compared with the average value from 2009 to 2019. There were 6,753,859 cases of class C infectious diseases in the two years, with an incidence rate of 239.2/100,000 people, a decrease of 17.32% compared with the average value from 2009 to 2019 (Table 3).

**Table 3:** The incidence change of notifiable infectious diseases comparing with in 2020–2021 and 2009–2019

Classify	Average incidence in 2020–2021		Average incidence in 2009—2019		Growth rate(%)
	Case	Rate(1/100,000)	Case	Rate(1/100,000)	
All	13,161,948	466.2	84,376,753	559.6	-16.70
Class A and B	6,408,089	227.0	40,754,404	270.3	-16.04
Class C	6,753,859	239.2	43,622,349	289.3	-17.32

### Incidence of notifiable infectious diseases by different transmission methods

From 2020 to 2021, the incidence rates of nationally notifiable infectious diseases with four transmission routes were intestinal infectious diseases (170.8/100,000), blood-borne and sexually transmitted infections (151.4/100,000), respiratory infectious diseases (138.3/100,000), and natural focal and insect-borne diseases (5.6/100,000). Compared with the incidence rates in 2009–2019, the incidence rates for the intestinal infectious diseases, respiratory infectious diseases and natural focal and insect-borne diseases all decreased in 2020–2021, and the incidence rates for the blood-borne and sexually transmitted diseases increased slightly in 2020 − 2011. (Table 4).

**Table 4:** Comparison of the incidence change of notifiable infectious diseases with different transmission routes between 2009–2019 and 2020–2021

Transmission route	Average incidence in 2020–2021			Average incidence in 2009—2019			Growth rate(%)
	Case	Rate(1/100,000)		Case		Rate(1/100,000)	
Intestinal infectious diseases	4,823,536		170.8	36,800,240	244.1		-30.01
Blood borne and sexually transmitted diseases	4,275,156		151.4	22,204,996	147.3		2.81
Respiratory infectious diseases	3,905,961		138.3	24,390,024	161.8		-14.48
Natural focal and insect borne diseases	157,236		5.6	975,627	6.5		-13.94

### ITS based on the ARIMA model

#### ITS-ARIMA model building of the three categories of notifiable infectious diseases in China from 2009 to 2020

**Fig. 2:**
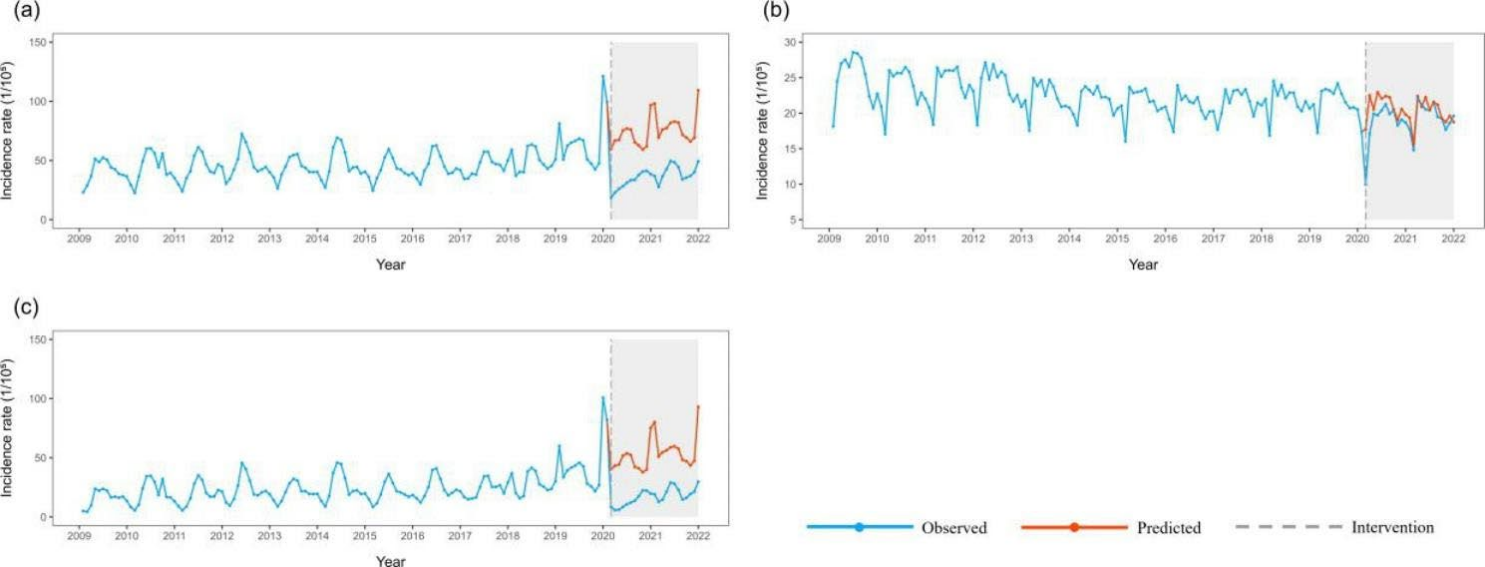
Incidence rates and counterfactual predictions of infectious diseases from three categories in China from 2009 to 2021 a:All; b:Classes A and B; c: Class C

After eliminating seasonal and long-term trends through the ARIMA model, the results showed that the incidence rates of the three categories of notifiable infectious diseases all decreased temporarily after the COVID-19 epidemic. The incidence rates of total, Classes A and B and Class C infectious diseases decreased by 39.791/100,000, 5.188/100,000, and 34.164/100,000, respectively. The incidence rates of total and Class C infectious diseases remained low in the long term. The incidence rates of Class A and B infectious diseases recovered to historical levels in the long term after the COVID-19 epidemic (step = 0.313) (Table 5; Fig. 2).

**Table 5:** ITS-ARIMA modeling of the incidence of three categories infectious diseases in China from 2009 to 2021

Classify	The optimal ITS-ARIMA model	*P* value Ljung-Box Test’s P value	Step	95%CI step	Ramp	95%CI Ramp
All	ARIMA(0,1,2)×(1,0,2)12	0.172	-39.791^*^	(-52.985, -26.596)	0.233	(-0.669, 1.134)
Class A and B	ARIMA(2,1,0)×(2,1,1)12	0.093	-5.188^*^	(-6.967, -3.409)	0.313^*^	(0.007, 0.620)
Class C	ARIMA(0,1,2)×(1,0,2)12	0.180	-34.164^*^	(-47.035, -21.293)	0.097	(-0.767, 0.097)

^*^ indicates that there is significantly difference

### ITS-ARIMA model of notifiable infectious diseases with different transmission routes in 2009–2021

**Fig. 3:**
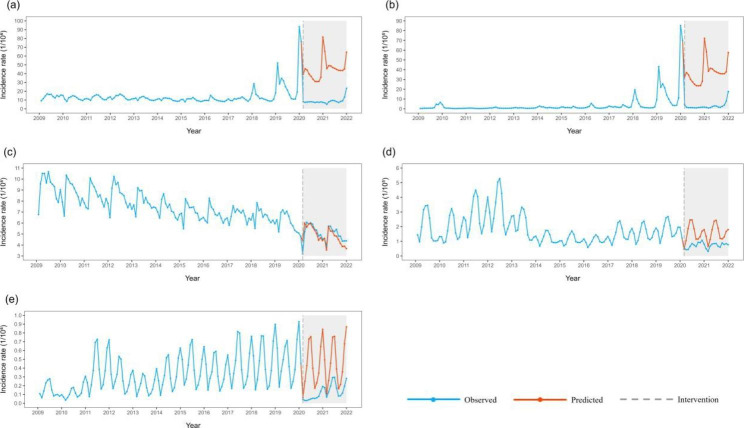
Incidence and counterfactual prediction of respiratory infectious diseases in China from 2009 to 2021 a: Respiratory infectious diseases; b: Influenza; c: Tuberculosis; d: Mumps; e: Scarlet fever

After the seasonal and long-term trends were eliminated by the ARIMA model, the changes in the incidence rates of infectious diseases with different transmission routes after the COVID-19 outbreak were as follows (Table 6):

Respiratory infectious diseases : The total incidence of respiratory infectious diseases showed an obvious short-term decrease after the COVID-19 outbreak (step =-29.828). The rate remained at a low level for a long time after the decline. Among the four major respiratory infectious diseases, the incidence of influenza and tuberculosis showed a significant short-term decline and remained at a low level after the decline. The incidence of scarlet fever and mumps did not change significantly before and after the COVID-19 epidemic (Fig. 3).

**Fig. 4:**
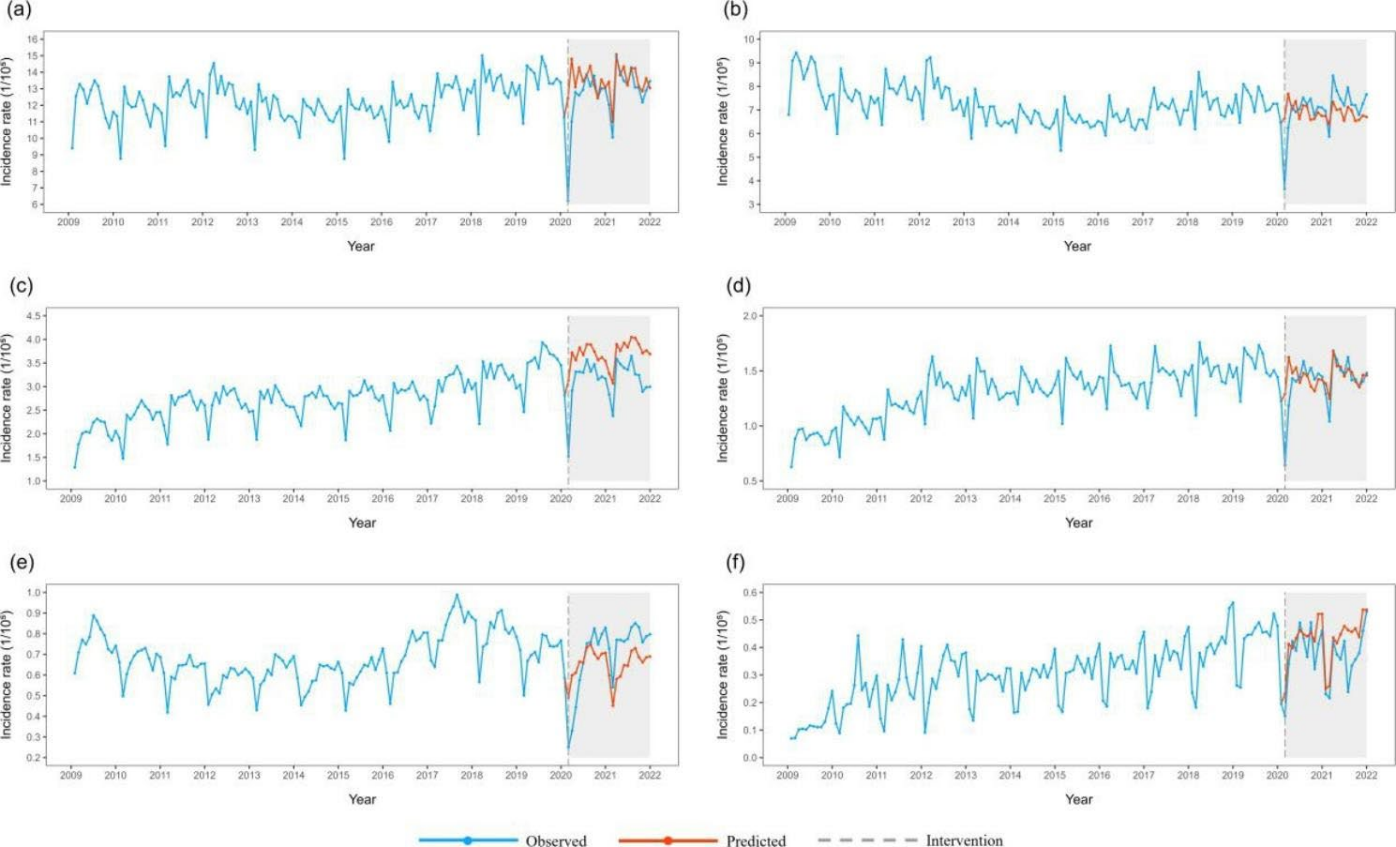
Incidence and counterfactual prediction of blood-borne and sexually transmitted infectious diseases in China from 2009 to 2021 a: Blood-borne and sexually transmitted diseases; b: Hepatitis B; c:Syphilis; d:Hepatitis C; e: Gonorrhoea; f: HIV/AIDS

Blood-borne and sexually transmitted infectious diseases: The total incidence of blood-borne and sexually transmitted infectious diseases (step =-3.638, ramp = 0.172) showed a short-term decline and recovery to historical levels in the long term. Among the main diseases, the incidence rates of hepatitis B, syphilis, and hepatitis C all decreased temporarily and remained at a low level after the decline. Then, the incidence of gonorrhoea decreased temporarily and had a long-term trend of recovery (Fig. 4).

**Fig. 5:**
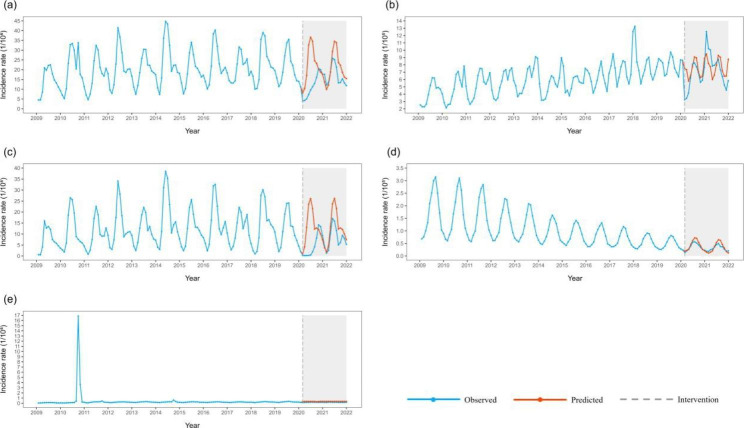
Incidence and counterfactual prediction of intestinal infectious diseases in China from 2009 to 2021 a: Intestinal infectious diseases; b: Other infectious diarrhoea; c: Hand, foot, and mouth disease; d: Dysentery; e: Acute haemorrhagic conjunctivitis

Intestinal infectious diseases: The total incidence of intestinal infectious diseases decreased temporarily (step = -8.367) and remained at a low level after the decline. Among the four major diseases, the incidence of other infectious diarrhoeal diseases decreased temporarily and remained at a low level after the decline; the incidence of hand, foot, and mouth disease, dysentery, and acute haemorrhagic conjunctivitis did not change significantly before and after the COVID-19 epidemic and before (Fig. 5).

**Fig. 6:**
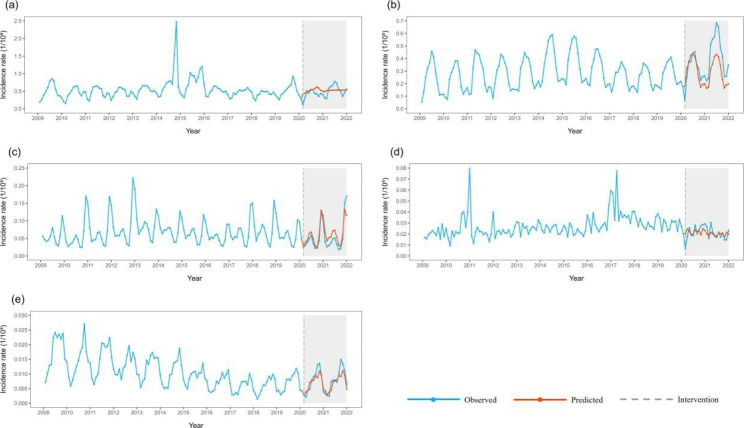
Incidence and counterfactual prediction of natural focal and insect-borne diseases in China from 2009 to 2021 a: Natural focal and insect-borne diseases; b: Brucellosis; c: Epidemic haemorrhagic fever; d: Echinococcosis; e: Typhus

Natural focal and insect-borne infectious diseases: The total incidence rates of natural focal and insect-borne infectious diseases did not change significantly before and after the COVID-19 epidemic. Among the four major diseases, only the incidence of brucellosis decreased temporarily and recovered to historical levels (Fig. 6).

**Table 6:** ITS-ARIMA modeling of the incidence of infectious diseases with different transmission routes in China from 2009 to 2021

Transmission routes	The optimal ITS-ARIMA model	*P* value Ljung-Box Test’s P value	Step	95%CI step	Ramp	95%CI Ramp	
Respiratory infectious diseases	ARIMA(0,1,2)×(0,0,2)12	0.317	-29.828^*^	(-44.364, -15.292)	-0.046	(-1.005, 0.913)	
Influenza	ARIMA(0,1,2)×(0,0,2)12	0.178	-27.704^*^	(-42.960, -12.447)	-0.041	(-1.027, 0.944)	
Tuberculosis	ARIMA(2,1,0)×(2,1,1)12	0.071	-0.653^*^	(-1.273, -0.032)	0.073	(-0.043, 0.190)	
Mumps	ARIMA(3,0,1)×(0,1,1)12	0.064	0.118	(-0.337, 0.573)	-0.03	(-0.103, 0.043)	
Scarlet fever	ARIMA(0,0,3)×(2,1,0)12	0.248	-0.122	(-0.276, 0.031)	-0.009	(-0.021, 0.003)	
Blood borne and sexually transmitted diseases	ARIMA(3,0,0)×(2,1,0)12	0.526	-3.638^*^	(-4.930, -2.346)	0.172^*^	(0.067, 0.277)	
Hepatitis B	ARIMA(2,1,0)×(2,0,0)12	0.617	-1.787^*^	(-2.597, -0.976)	0.132	(-0.001, 0.265)	
Human immunodeficiency virus/acquired immunodeficiency Syndrome (HIV/AIDS)	ARIMA(0,1,4)×(0,1,1) 12	0.731	-0.048	(-0.113, 0.017)	-0.002	(-0.008, 0.003)	
Syphilis	ARIMA(2,1,1)×(0,1,1)12	0.222	-0.772^*^	(-1.052, -0.492)	0.008	(-0.025, 0.041)	
Hepatitis C	ARIMA(2,1,0)×(0,1,1)12	0.292	-0.416^*^	(-0.574, -0.258)	0.024	(-0.001, 0.049)	
Gonorrhoea	ARIMA(0,1,2)×(1,1,1)12	0.186	-0.285^*^	(-0.371, -0.199)	0.017^*^	(0.003, 0.03)	
Intestinal infectious diseases	ARIMA(2,0,0)×(2,1,1)12	0.827	-8.367^*^	(-14.921, -1.813)	0.198	(-0.277, 0.673)	
Other infectious diarrhoea	ARIMA(0,0,2)×(0,1,2)12	0.998	-2.331^*^	(-3.844, -0.818)	0.062	(-0.046, 0.17)	
Hand-foot- mouth disease	ARIMA(1,0,1)×(0,1,1)12	0.626	-3.850	(-9.933, 2.233)	-0.018	(-0.489, 0.454)	
Dysentery	ARIMA(2,1,2)×(0,1,1)12	0.001	-0.077	(-0.165, 0.010)	0.004	(-0.005, 0.012)	
Acute haemorrhagic conjunctivitis	ARIMA(1,0,0)×(2,0,2)12	1.000	-0.170	(-1.565, 1.225)	-0.001	(-0.100, 0.099)	
Natural focal and insect borne diseases	ARIMA(2,0,1)×(0,0,1)12	0.774	-0.244	(-0.534, 0.047)	0.013	(-0.007, 0.034)	
Brucellosis	ARIMA(1,0,1)×(0,1,1)12	0.059	-0.113^*^	(-0.185, -0.041)	0.013	(0.006, 0.019)	
Epidemic haemorrhagic fever	ARIMA(3,0,0)×(0,1,1)12	0.983	-0.004	(-0.027, 0.019)	0.001	(-0.001, 0.003)	
Echinococcosis	ARIMA(0,1,2)×(2,0,0)12	0.759	-0.006	(-0.017, 0.006)	0.001	(-0.001, 0.002)	
Typhus	ARIMA(0,1,2)×(2,1,1)12	0.550	-0.001	(-0.003, 0.003)	0.001	(-0.001, 0.001)	

^*^ indicates that there is significantly difference

## Discussion

In this study, there was a significant decline in the incidence rates of infectious diseases after the COVID-19 epidemic comparing before the epidemic, especially respiratory and enteric infectious diseases, which remained low for a long time after the decline. As early as 2008, China approved a major scientific and technological project for the “Prevention and Control of Major Infectious Diseases such as AIDS and Viral Hepatitis“[16], which aimed to improve the national scientific and technological support system for the prevention and control of infectious diseases and improve the level of diagnosis, prevention and treatment of infectious diseases. For more than a decade, the incidence of reported infectious diseases has shown a generally stable trend [17]. With the outbreak of COVID-19 in 2020, all provinces in China implemented an emergency response, and a series of prevention and control measures were adopted to control the epidemic. These measures also had a considerable impact on other infectious diseases, such as measles, pertussis, scarlet fever, seasonal influenza, and mumps[3, 4].

This study showed that the incidence of notifiable infectious diseases in China declined significantly for a short time, especially for the Class C infectious diseases. Main reason was likely that the incidence of influenza and other infectious diarrhoeal diseases, accounted for a large proportion of Class C infectious diseases, and the incidence of these diseases declined significantly. Children and adolescents are susceptible to influenza and other infectious diarrhoeal diseases[18, 19]. School suspension measures may have affected the incidence rate of these disease in the early stage of the COVID-19 epidemic. Moreover, the incidence rates of some infectious diseases remained low for a long time, which showed that prevention and control measures for COVID-19 improved healthy behaviours, and had long-term preventive and control effects on infectious diseases. After May 2020, with the amelioration of the domestic epidemic, most provinces modified or cancelled their emergency response measures and entered a normal stage.

Respiratory infectious diseases were substantially affected by the COVID-19 epidemic. One of the reasons may be that COVID-19 is a respiratory infectious disease, and prevention and control measures for COVID-19 were similarly effective for other respiratory infectious diseases[20]. The measures included cutting off the transmission route of the COVID-19 virus by strengthening ventilation and personal protection (wearing a mask and hand hygiene) and reducing the number of gatherings. Second, children and adolescents are susceptible to influenza. In the early stage of the epidemic, schools took measures including halting offline teaching, which reduced gatherings among people and caused a temporary and substantial decrease in the incidence of respiratory infectious diseases. Third, some studies have shown that there is a “virus interference phenomenon” among respiratory viruses. The infection of one virus can partially prevent or inhibit the infection of another virus in the same host [21]. Some studies have proposed that the decline in the incidence of influenza after COVID-19 may be due to this “virus interference phenomenon [22]. Fourth, research has shown that after the COVID-19 epidemic, the public had a high level of knowledge and awareness of the prevention and control of respiratory infectious diseases [23, 24]. In addition, studies have also shown that after the COVID-19 epidemic, the public adopted healthy behaviours more actively, such as covering the mouth and nose while coughing and sneezing, keeping the room clean, ventilating frequently, and avoiding gatherings [25]. The public paid more attention to personal protection, and took the initiative to adopt healthy behaviours, which greatly reduced the incidence of respiratory infectious diseases, which remained low after a short period of substantial decline.

The incidence of blood-borne and sexually transmitted diseases showed a short-term decline and an upward trend in a long time. The main blood-borne diseases in China are viral hepatitis including hepatitis B, hepatitis C and sexually transmitted diseases (STDs). Chronic hepatitis has a long incubation period and mild symptoms during the incubation period. Most patients are diagnosed with hepatitis B at an early stage through hepatitis B screening and physical examinations. During the early stage of the COVID-19 epidemic, most hepatitis B screening programs were postponed due to the need for epidemic prevention, and residents’ medical needs were likely suppressed [26], which resulted in a short-term decrease in the incidence of blood-borne and sexually transmitted diseases. At the late stage of the COVID-19 epidemic, work and school all returned to norma with the improvement of the epidemic situation, and the demand for medical treatment changed. The incidence of blood-borne and sexually transmitted diseases recovered to the usual level before the COVID-19 pandemic.

The incidence rate of intestinal infectious diseases showed a short-term decline after the COVID-19 epidemic, and remained for a long time. Intestinal infectious diseases are mainly transmitted by daily contact [27]. During the COVID-19 epidemic, online teaching may have reduced student gatherings and directly stopped the spread of infectious diseases. Moreover, many collective units, such as schools, strengthened disinfection and cleaning during the COVID-19 epidemic [28], which greatly reduced the number of infectious caused by intestinal infectious diseases through daily contact. This healthy behaviour played a role in long-term prevention and control measures for intestinal infectious diseases. Studies have shown that health education for primary and secondary school students after the COVID-19 epidemic improved the group’s awareness of hand hygiene and other healthy behaviours, and students pay more attention to hygiene habits and correct handwashing methods[29] .

Among natural focal and insect-borne infectious diseases, only the incidence rate of brucellosis was temporarily decreased. This may be because the incidence of brucellosis is mostly concentrated in relevant occupational groups, such as animal husbandry, the breeding industry, the slaughtering industry and herdsmen[30]. The main reason for the decline was likely the measures taken to stop work during the COVID-19 epidemic.

### Limitations and strengths

The strengths of this study were its use of nationally notifiable infectious disease data from more than ten years in China, ITS to control for the impact of seasonality and cyclicity on the incidence rate of infectious diseases, and its analysis of the impact of measures for COVID-19 epidemic prevention and control on other infectious diseases during the COVID-19 pandemic. The research data source is reliable, and the method is scientific, and the conclusion is meaningful. The limitation of this study was that the collection period after the COVID-19 epidemic was relatively short. Moreover, policies change quickly. It is necessary to conduct futher research in the future.

Ethics Approval and Consent to Participate.

The ethics committee of the Guangzhou Center of Disease Control and Prevention approved this study proposal. This study used public data to establish a mathematical model, and didn’t involve individual survey and personal details.

## Conclusion

This study showed that the prevention and control measures implemented during the COVID-19 epidemic had a large short-term and long-term impact on most of the nationally notifiable infectious diseases in China, and mainly impacted respiratory infectious diseases and intestinal infectious diseases. There were also short-term prevention and control effects on blood-borne and sexually transmitted infectious diseases.

This study provides evidence for the prevention and control of infectious diseases in the future. The measures of COVID-19’s prevention and control, such as wearing masks, hand hygiene and reducing interpersonal distance, are also effective for respiratory and intestinal infectious diseases such as influenza, tuberculosis, other infectious diarrhoeal diseases and syphilis.

## Data Availability

The datasets generated during analyses are available in the journal of “Disease surveillance”. http://www.jbjc.org/index.htm.

## Abbreviations

COVID-19: Novel coronavirus pneumonia
ITS: Interrupted time series
ARIMA: Autoregressive integrated moving average
STDs: Sexually transmitted diseases
SARS: Severe acute respiratory syndrome
HIV/AIDS: Human immunodeficiency virus/acquired immunodeficiency syndrome
